# Are choriocapillaris flow void features robust to diurnal variations? A swept-source optical coherence tomography angiography (OCTA) study

**DOI:** 10.1038/s41598-020-68204-x

**Published:** 2020-07-09

**Authors:** Emily Lin, Mengyuan Ke, Bingyao Tan, Xinwen Yao, Damon Wong, Lirong Ong, Leopold Schmetterer, Jacqueline Chua

**Affiliations:** 1 Singapore Eye Research Institute, Singapore National Eye Centre, 20 College Road, The Academia, Level 6, Discovery Tower, Singapore, 169856 Singapore; 2 SERI-NTU Advanced Ocular Engineering (STANCE), Singapore, Singapore; 3 Institute for Health Technologies, Nanyang Technological University, Singapore, Singapore; 4 Department of Clinical Pharmacology, Medical University Vienna, Vienna, Austria; 5 Center for Medical Physics and Biomedical Engineering, Medical University Vienna, Vienna, Austria; 6 Institute of Molecular and Clinical Ophthalmology, Basel, Switzerland; 7 Ophthalmology and Visual Sciences Academic Clinical Program, Duke-NUS Medical School, Singapore, Singapore

**Keywords:** Medical imaging, Diagnostic markers

## Abstract

We evaluated the impact of diurnal variation on choroidal and retinal microvasculature and structural measurements using a swept-source optical coherence tomography angiography machine (SS-OCTA; PLEX Elite 9,000, Carl Zeiss Meditec, Inc., Dublin, USA). Fourteen participants who were without ocular diseases underwent SS-OCTA imaging using 3 × 3-mm^2^ macular scan pattern on two separate days at five time points. Choriocapillaris flow voids were generated to determine its density (percentage), size (μm) and numbers. Perfusion densities of the large superficial vessels, as well as capillaries on superficial and deep vascular plexuses were generated from retinal angiograms. Subfoveal choroidal and retinal thicknesses were manually measured. Repeated-measures ANOVA was used to investigate the impact of diurnal variation on choroidal and retinal measurements. There was no observable diurnal pattern for any of the flow void features, in terms of the density, size and numbers. There was a significant diurnal pattern observed in the choroidal thickness, where it decreased progressively during the day (P < 0.005). As opposed to sub-foveal choroidal thickness, there does not appear to be significant diurnal variation in choriocapillaris flow voids in normal individuals. This suggests that alterations of choriocapillaris flow deficit seen in pathological eyes will not be confounded by the diurnal fluctuation.

## Introduction

There is increasing evidence that the alterations of choroidal blood flow play a crucial role in various ocular diseases^1^ like age-related macular degeneration (AMD)^2^, pathological myopia^3^, and diabetic retinopathy (DR)^4^. These studies have postulated that a decreased in the choroidal blood perfusion may be reflected by a thinning of the choroid^2–4^. However, using choroidal thickness as a biomarker of the choroidal blood flow may not be an optimal choice because, first, it is not a direct measure of blood flow; second, it is not robust enough because it notoriously suffers from diurnal variability^5,6^.

Swept-source optical coherence tomography angiography (SS-OCTA) is an imaging tool that can acquire three-dimensional angiograms non-invasively^7,8^. It provides tissue imaging for deeper choroidal vasculature, while at the same time offering important insights to retinal/choroidal disorders^9^. Of note, OCTA is limited, and that flows below a particular threshold are undetectable from background noise and are thus several terms such as signal void^10^ or flow voids have been used to described the choriocapillaris nonperfusion or flow impairment. Studies have used SS-OCTA to quantify flow voids within the choriocapillaris in patients with hypertension^11^, and a variety of eye diseases^9,12^ and non-human primates^13^. However, it remains unclear if these newly proposed choriocapillaris flow void measurements are affected by diurnal change. Having a vascular imaging biomarker of the choroid that is impervious to diurnal variation may be clinically meaningful for the monitoring of eye diseases.

Recent studies have examined the impact of diurnal variations on choriocapillaris flow voids but yielded controversial results^14–16^. Sarwar et al. observed a significant decrease in choriocapillaris flow density over two time points while Rommel et al. did not observe a diurnal variation in the choriocapillaris perfusion over four time points. However, both have used the spectral domain optical coherence tomography (SD-OCTA) system where the instrument’s wavelength of 840 nm may make it difficult to image the choriocapillaris^14–16^. In contrast, SS-OCTA has a longer wavelength and less sensitivity roll-off, allowing for an improved imaging speed and penetration that may benefit the visualization of the choriocapillaris^13^. Therefore, the SS-OCTA, with the various advantages over SD-OCTA, should serve as a better system for choriocapillaris imaging. In this study, we investigated the effect of diurnal variation on the quantitative choriocapillaris microvasculature (density, size and numbers) using SS-OCTA (PLEX Elite 9,000, Carl Zeiss Meditec, Inc., Dublin, USA; Version 1.7) and compared this to variations of choroidal thickness in healthy volunteers. A further aim was to investigate if retinal microvasculature and structural measurements were robust to diurnal variations.

## Results

A total of 14 eyes from 14 participants with good scans were included in the current analysis. We excluded one participant due to segmentation errors which were a result of poor image quality. The right eye of each participant was chosen for this study, unless the image quality was not ideal for any of the 5 time points. If this was the case, the left eye was chosen instead. The participants were 31 ± 6 years old, ranging from 22 to 47, of which two-thirds were female and 79% were Chinese (n = 11). They did not have any history of systemic and ocular diseases or surgeries. Their spherical equivalent was − 3.82 ± 2.79 D. OCTA scans had a mean signal strength of 9.91 ± 0.28. There was excellent inter-grader (ICC = 0.99) and intra-grader (first grader; ICC = 0.98 and second grader; ICC = 0.99) agreements in terms of sub-foveal choroidal and retinal thickness measurements.

**Figure 1:**
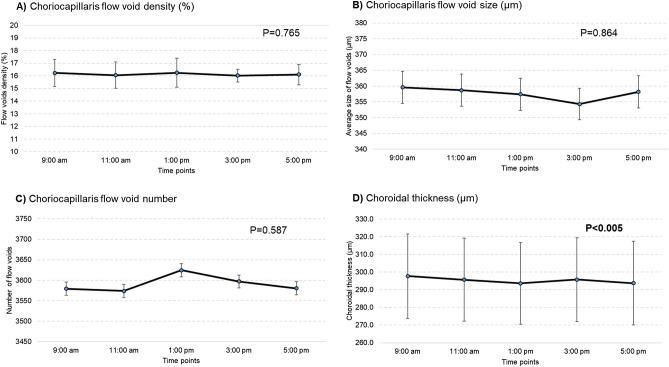
There was no diurnal variation on any of the choriocapillaris flow voids features (**A**–**C**; P > 0.05). Diurnal variation had an impact on choroidal thickness, where it was thickest in the morning and thinnest in the evening (**D**; P < 0.005). P values were obtained with repeated-measures ANOVA, where within-subject factor (time of visit) was adjusted.

**Figure 2:**
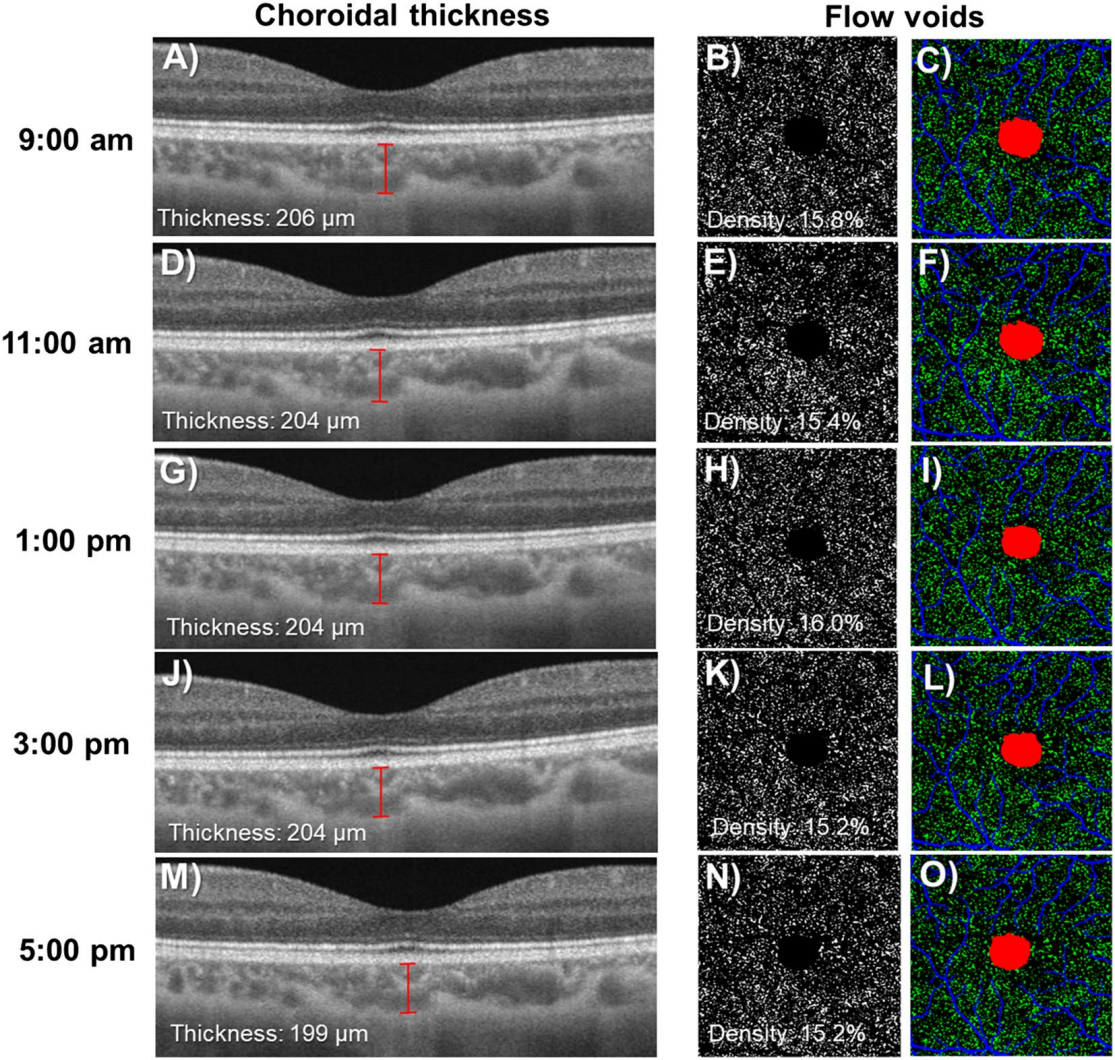
Choroidal thicknesses (**A**, **D**, **G**, **J**, and **M**) decreased throughout the day whereas the density of flow voids as seen in the binarized (**B**, **E**, **H**, **K**, and **N**) and color-coded (**C**, **F**, **I**, **L**, and **O**) images appeared consistent. Images A, D, G, J, and M were generated from the built-in review software (PLEX Elite Review Software, Carl Zeiss Meditec, Inc., Dublin, USA; Version 1.7.1.31492; https://www.zeiss.fr/content/dam/Meditec/international/ifu/documents/plex-elite/current/2660021169042_rev._a_artwork.pdf).

Table 1 shows the change in choriocapillaris flow voids and choroidal thickness throughout the day. The features of choriocapillaris flow voids did not change significantly over time on the first visit, where the density was 16.2 ± 1.1%, average size of flow void was 359.6 ± 26.6 µm and number was 3,579.4 ± 151.5 (P > 0.05; Table 1 and Fig. 1A–C). These findings remained similar for the second visit. In terms of choroidal thickness, there was a small but statistically significant decrease in the thickness over time (Table 1 and Fig. 1D). On average, the choroid was thickest at 9:00 am at 297.7 ± 92.1 µm, followed by 295.7 ± 91.0 µm at 11:00 am, 293.6 ± 89.5 µm at 1:00 pm, 295.7 ± 91.8 µm at 3:00 pm and thinnest 293.8 ± 91.5 µm at 5:00 pm (P < 0.005) for the first visit. These findings remained similar for the second visit (P < 0.002). Figure 2 shows an example of a patient where the thickness of the choroid was impacted by diurnal variation, while the density of the choriocapillaris flow voids remained unaffected.

**Table 1:** Changes in choroidal measurements over several time points and two visits.

Time points	9:00 am	11:00 am	1:00 pm	3:00 pm	5:00 pm	P value
**First visit measurement**						
*Choriocapillaris flow voids*						
Area (%)	16.2 ± 1.1	16.1 ± 1.0	16.3 ± 1.1	16.0 ± 0.5	16.1 ± 0.8	0.765
Size (µm)	359.6 ± 26.6	358.7 ± 33.2	357.4 ± 37.9	354.3 ± 22.2	358.2 ± 24.0	0.864
Numbers	3,579.4 ± 151.5	3,573.9 ± 254.1	3,624.4 ± 160.6	3,596.9 ± 186.3	3,590.3 ± 143.9	0.587
Choroidal thickness (µm)	297.7 ± 92.1	295.7 ± 91.0	293.6 ± 89.5	295.7 ± 91.8	293.8 ± 91.5	**0.005**
**Second visit measurement**						
*Choriocapillaris flow voids*						
Area (%)	16.3 ± 0.9	16.5 ± 1.1	15.9 ± 0.7	16.0 ± 0.6	16.1 ± 0.5	0.099
Size (µm)	357.0 ± 27.3	364.0 ± 36.5	350.3 ± 23.2	350.8 ± 25.7	353.4 ± 17.5	0.242
Numbers	3,615.8 ± 113.5	3,617.7 ± 144.8	3,616.0 ± 168.3	3,624.0 ± 143.0	3,633.6 ± 130.6	0.947
Choroidal thickness (µm)	296 ± 95.5	295.2 ± 93.8	293.4 ± 94.4	292.8 ± 93.8	292 ± 94.4	**0.002**

Data presented are in mean ± SD.

Bold values denote statistical significance at the P < 0.05 level.

Table 2 shows the change in retinal perfusion densities and retinal thickness throughout the day. There was no influence of diurnal variation on any of the retinal microvascular and thickness measurements on the first visit, where the large perfusion density ranged in 9.6–10.0%, superficial perfusion density 27.3–28.1%, deep perfusion density was 15.2–15.5% and retinal thickness was 221.9–222.7 µm (P > 0.05; Table 2). These findings remained similar for the second visit.

**Table 2:** Changes in retinal measurements over several time points and two visits.

Time points	9:00 am	11:00 am	1:00 pm	3:00 pm	5:00 pm	P value
**First visit measurement**						
*Retinal perfusion densities*						
Large vessels (%)	10.0 ± 1.3	9.6 ± 1.4	9.8 ± 1.8	9.8 ± 1.3	9.7 ± 1.5	0.690
Superficial perfusion density (%)	28.1 ± 2.2	27.7 ± 2.5	27.3 ± 2.3	27.9 ± 2.2	27.6 ± 2.7	0.231
Deep perfusion density (%)	15.5 ± 1.6	15.2 ± 1.9	15.3 ± 1.6	15.5 ± 1.6	15.4 ± 1.7	0.647
Retinal thickness (µm)	222.4 ± 28.7	221.1 ± 29.6	222.7 ± 28.8	221.6 ± 30.1	221.9 ± 29.1	0.325
**Second visit measurement**						
*Retinal perfusion densities*						
Large vessels (%)	10.0 ± 1.7	9.4 ± 1.6	9.6 ± 1.4	9.7 ± 1.5	9.5 ± 1.2	0.224
Superficial perfusion density (%)	27.7 ± 2.6	26.6 ± 2.7	27.0 ± 2.5	27.2 ± 2.7	27.3 ± 2.3	0.430
Deep perfusion density (%)	15.3 ± 1.5	14.8 ± 2.0	15.2 ± 1.8	15.6 ± 1.7	15.4 ± 1.4	0.386
Retinal thickness (µm)	221.6 ± 28.61	221.7 ± 29.1	222.7 ± 29.9	222.1 ± 30.3	222.1 ± 29.6	0.493

Data presented are in mean ± SD.

## Discussions

In this SS-OCTA based study, we found that the features of choriocapillaris flow void were unaffected by diurnal variation whereas as expected, the thickness of the choroid was impacted by diurnal variation in healthy volunteers without eye or systemic diseases. Retinal vasculature and structural measurements, such as perfusion densities of large vessels, superficial and deep vascular plexuses and retinal thickness, were not subjected to diurnal variation. Our results suggest that the SS-OCTA derived choriocapillaris flow voids measurement used currently in clinical studies will not be prejudiced by diurnal changes.

Quantification of choriocapillaris flow voids has gained considerable attention. AMD studies have demonstrated an increased average choriocapillaris flow void size in eyes with unilateral Type 3 neovascularization than fellow non-neovascular eyes^17^. Additionally, intermediate AMD eyes of patients with neovascular AMD in the fellow eye had choriocapillaris signal void that was larger in size than eyes who without neovascular AMD in the fellow eye^10^. Those intermediate AMD eyes that demonstrated increased CC flow impairment had an area of choriocapillaris flow void that co-localized with a nearby surrounding drusen^18^. Apart from eye diseases, blood pressure measurements have been shown to alter the choriocapillaris flow void patterns in patients with systemic hypertension, which further suggests the potential role of choriocapillaris flow void mapping in systemic diseases^11^. Given the robustness of the choriocapillaris flow voids to diurnal variation, the evaluation of choriocapillaris flow voids may enhance the understanding of the pathophysiology of AMD.

Recent studies have examined the impact of diurnal variations on choriocapillaris flow voids using OCTA in normal subjects^15,16,19^ and patients with idiopathic epiretinal membrane^14^. In a study of 25 normal individuals^15^, SD-OCTA scans were performed at two time points with the AngioVue (Optovue, Fremont, CA, USA). The authors found a significant decrease in the choriocapillaris flow density. However, a difference between two time points is less convincing than if the measurements were performed at several additional points during the day. Another study performed choriocapillaris flow voids measurements using the HS-100 OCTA (Canon, Tokyo, Japan) over four time points in 22 normal subjects^14^. The authors found no significant variation in the choriocapillaris flow density measurements over the four time points, which agrees with our study. There is, however, common limitations in the aforementioned studies: they have used a SD-OCTA, which has a shorter wavelength and conducted their measurements only on a single visit^14^. In comparison, we used the SS-OCTA which uses a longer wavelength and its reduced sensitivity roll-off, resulting in enhanced light penetration through the RPE, as well as better detection of signals from the deeper layers^20^. Furthermore, our examination sequence (five OCTA scans during the day) was repeated on a separate day to confirm the consistency of the results. We provided a more comprehensive assessment of the flow voids and demonstrated that the average size and number of the flow voids remained unaffected by diurnal variation.

Previous studies have investigated the potential diurnal variations of choroidal blood flow^21–25^. Using laser speckle flowgraphy, diurnal variations of optic nerve head and choroidal blood flow were reported with a trough at 9:00 h and a peak at 24:00 h and a trough at 15:00 h and a peak at 18:00 h, respectively^21^. The same group also reported diurnal variation in the waveform of the laser speckle flowgraphy waveforms^22^. By contrast, others have employed different techniques such as laser Doppler flowmetry, color Doppler imaging, laser interferometric measurement of fundus pulsation and did not observe diurnal variations of blood flow parameters^23–25^.

The choroidal thickness is unlikely to be an ideal indicator of disease progression monitoring because of the mounting evidence that it is subject to diurnal variation. Previously, studies have reported the diurnal fluctuation in the choroidal thickness, ranging from 12 to 40 μm^5, 6,16,26,27^. Tan et al. used the Spectralis SD-OCT (Heidelberg Engineering) with an axial resolution of 3.9 μm observed in 12 healthy volunteers a mean diurnal variation of 34 μm, where the choroid was found to be thickest in the morning and thinner in the evening^5^. In another study of 24 young subjects, significant diurnal variations of 29 μm were observed, but controversially they found the choroid to be thinnest in the morning and thicker at night^6^. It may be that they have used an optical biometer, which may not be comparable to OCT. Usui et al. used a swept source prototype with an axial resolution was 8 μm and also reported a mean amplitude of approximately 33 µm^26^ and Siegfried et al. used the SD-OCT HS-100 OCTA (Canon, Tokyo, Japan) with an axial resolution was 1.6 μm reported a mean amplitude of approximately 40 µm^16^. Gabriel et al. used a SD-OCT with a wavelength of 1,060 nm with an axial resolution of 7 μm and also reported a small amplitude change of approximately 12 µm^27^. In our study, we used the PLEX Elite 9,000 with an axial resolution of 6.8 μm and observed a small but statistical significance amplitude change in choroidal thickness of 8 µm. It is difficult to fully explain the small change in choroidal thickness seen in our study compared to others. This discrepancy may be due to a mix of different factors, including the time span of the experiment. For example, our study examined patients from 9 am to 5 pm while Usui covered 24 h^26^. Other factors pertaining to the hardware of the OCT systems include the detection precisions of different OCT having different axial resolutions, central wavelengths of 840 nm versus 1,060 nm, and scanning speeds. Human factors such as refractive error and axial length are known to be correlated with the amplitude of choroidal thickness variation^5^. As choroidal thickness was only measured at a single measurement at the fovea pit, we can safely rule out the effect of OCT magnification. Tan et al. found that those with axial length of 23.5 mm or shorter tended to exhibit larger variation. In our study, 86% of our subjects had axial length longer than 23.5 mm, which may explain the small variation. For studies that have decided to use choroidal thickness as a biomarker for choroidal perfusion, they will need to take into consideration the effect of diurnal fluctuation. Also, as a result of diurnal changes, the choroidal thickness measurement will be more variable, which will increase the confidence intervals of measurements and may reduce the precision of the data.

It should be noted that we and others have measured the sub-foveal choroidal thickness, which is limited to a single measurement underneath the fovea^5,6,16,26,27^. The choroid is highly vascularized and is composed of an anastomosed network of capillaries^1^. Single readings may potentially be a significant source of error as they may not offer a complete representation about changes in the entire choroid. Shin and co-workers proposed a novel technique where vast amounts of data over a large area of choroid were used to create a topographic choroidal thickness map, which may provide a more comprehensive assessment of the choroid^28^. Nevertheless, choroidal vascular perfusion would probably be better assessed using the flow voids map than the choroidal thickness. The former may be directly linked to the oxygenation of the outer retina whereas the latter may show diurnal thickness changes that are independent of vascular perfusion. Data relating choroidal thickness to the perfusion status of the choroid and the oxygenation of the outer retina are currently lacking.

We would like to highlight the difference in the vascular density in our study as compared to previous publications. In the current study, the vascular density of retinal layer ranged in 27.0–28.1% of superficial layer, 14.8–15.6% of deep layer. This is different from previous publications, where they demonstrated it as between 40–50% in normal subjects^29,30^. We would like to offer a few potential reasons. First, different post-processing algorithms can have substantial effect on OCTA metrics, including decorrelative signal generation, vessel enhancement and segmentation. Second, different hardware and scanning protocols can result in a difference in the OCTA metrics, such as scanning speed, lateral resolution, numbers of A-scan and B-scan. We have shown that using different filters can generate perfusion density, ranging from 29.8 to 45.1% in a 3 × 3 mm^2^ macula scan^31^. Therefore, OCTA metrics are not comparable without considering these factors. Nevertheless, some other studies reported similar perfusion density values, using Frangi filter^32,33^. Thus far, there is no consensus on which filter and which parameters are optimal for extracting the OCTA metrics. We have shown previously high perfusion density reproducibility using frangi filter^31^, and the parameters were tuned so that the binarized perfusion map has highest similarity to anatomy.

Our present study has a few limitations. First, the relatively small sample size to make definitive conclusive that choriocapillaris flow void measurements are impervious to diurnal changes. Second, all our participants had myopic prescription, where 71% had low-moderate myopia (− 0.25D to − 5D) while the rest had high myopia (− 5.50D to − 10.25D). It has been shown previously that myopic eyes tended to have more flow voids^34^. In particular, the choriocapillaris flow were more reduced within the foveal area, than the para- or peri-foveal regions^34^. Nevertheless, we excluded the foveal area from the analysis. Also, our myopic participants were free from myopic pathological changes such as chorioretinal atrophy^35^. The analysis was only based on measurements acquired during the day and measurements were not conducted during the night. This study included only normal individuals of a relatively small cohort of working-aged adults, which makes the results not generalizable for older persons.

In conclusion, we have found that choriocapillaris flow void features measured using the SS-OCTA were robust to diurnal variations in normal persons, highlighting that the alterations seen in SS-OCTA derived choriocapillaris flow voids in ocular diseases is not confounded by diurnal variations.

## Methods

### Study participants

We conducted a prospective study from June 2019 to August 2019 on 15 healthy volunteers, aged 21 and above, with no history of systemic or ocular diseases at the Singapore Eye Research Institute Clinic. All individuals were screened for suitability before recruitment. Ethics approval was obtained from the SingHealth Centralized Institutional Review Board. Written, informed consent was obtained for all participants in adherence to the Declaration of Helsinki.

### Examination procedures

The detailed study design and procedures performed as shown in Supplementary Table S1. Detailed interviewer-administered questionnaire was used to collect demographic data, lifestyle risk factors (e.g. smoking), medical history (e.g. diabetes, hypertension, high cholesterol) and ocular history (e.g. glaucoma, retinopathies or any surgery or laser treatment)^36,37^. Systolic and diastolic blood pressures (SBP, DBP) were measured using a digital automatic blood pressure monitor (Dinamap model Pro Series DP110X-RW, GE Medical Systems Information Technologies, Inc., Milwaukee)^36^. Participants were assessed for their axial length using non-contact partial coherence interferometry (IOL Master V3.01, Carl Zeiss Meditec AG, Jena, Germany), refractive error using an auto-refractor (Canon RK-5 Autorefractor Keratometer; Canon Inc., Tokyo, Japan) and intra-ocular pressure using airpuff tonometer^36^. Their pupils were dilated with 1% tropicamide and 2.5% phenylephrine hydrochloride before they underwent fundus photography and SS-OCTA. Fundus photographs further documented the absence of ocular diseases. Recruited participants consumed their daily dietary without any alteration throughout the course of study.

### Swept-source optical coherence tomography angiography imaging

All participants were scanned by the same trained ophthalmic technician (E.L). Both eyes of each participant were imaged using the Swept-Source Optical Coherence Tomography Angiography (SS-OCTA; PLEX Elite 9,000, Carl Zeiss Meditec, Inc., Dublin, USA; Version 1.7). Each participant received a single 3 × 3-mm^2^ macula centered scan at five different time points (9:00 am, 11:00 am, 1:00 pm, 3:00 pm and 5:00 pm) during the day spaced at 2-h intervals. A repeated series of scans was then performed on a different day (but at the same time points) at a week later. This device is a swept source OCT with eye tracking using FastTrac motion correction software and it operates at 1,060 nm central wavelength and at 100,000 A-scans per second with axial resolution of 6.3 µm and transverse resolution of 20 µm in tissue to acquire volumetric scans. Its A-scan depth penetration in tissue was 3.0 mm and the machine included an enhanced depth imaging (EDI) function. The OCTA machine provided a signal strength index, ranging from 0 to 10, where only images with a scan quality of 8 and above were accepted. Scans were retaken if there were significant motion artifacts, misalignment, poor contrast or uneven illumination.

#### Evaluation of retinal and choroidal thickness

**Figure 3:**
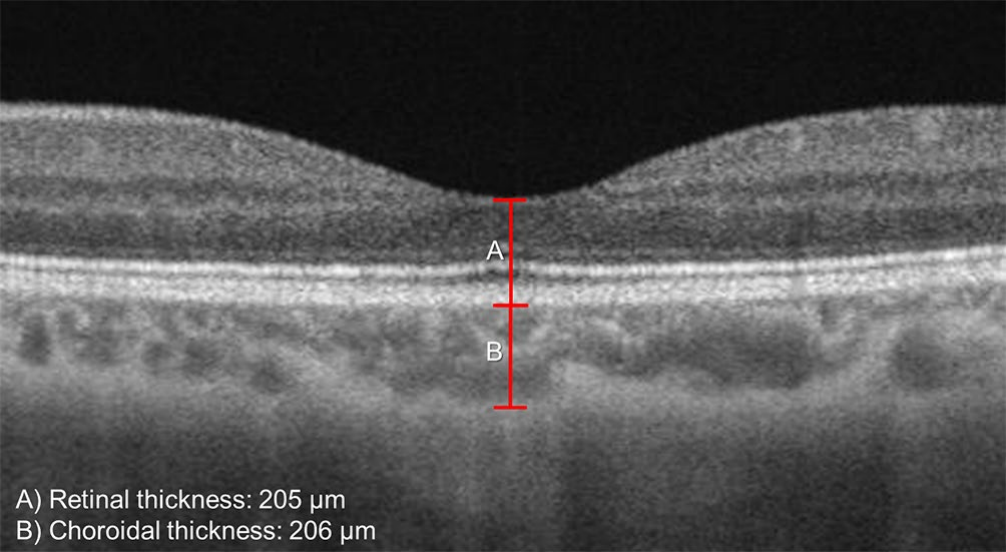
The retinal (**A**) and choroidal (**B**) thicknesses, were manually measured at the fovea (detected as the lowest point of internal limiting membrane), using caliper measurement tool. The images were generated from the built-in review software (PLEX Elite Review Software, Carl Zeiss Meditec, Inc., Dublin, USA; Version 1.7.1.31492; https://www.zeiss.fr/content/dam/Meditec/international/ifu/documents/plex-elite/current/2660021169042_rev._a_artwork.pdf).

Two independent graders who were blinded to the scan timing, performed the measurements for retinal and subfoveal choroidal thicknesses from the 3 × 3-mm^2^ macula centered scan. The thicknesses were measured at the center of the fovea using MATLAB (MathWorks, MA, USA). The built-in review software (PLEX Elite Review Software, Carl Zeiss Meditec, Inc., Dublin, USA; Version 1.7.1.31492) provided the retinal thickness map. To determine the foveal pit, we scanned through several B-scans to determine the thinnest retina layer. The neighboring two B-scans were averaged for segmentation of the choroidal-scleral boundary. The retinal thickness was marked from the inner limiting membrane (ILM) perpendicular to the outer surface of the retinal pigment epithelium (RPE) whereas the choroidal thickness was marked from the outer surface of the RPE to the CSI (Fig. 3). The thicknesses were measured with the caliper function in the MATLAB presented in pixel, and subsequently converted to micrometers via an axial digital sampling of 1.95 µm/ pixel.

### Evaluation of the retinal vasculature and the choriocapillaris flow voids

**Figure 4:**
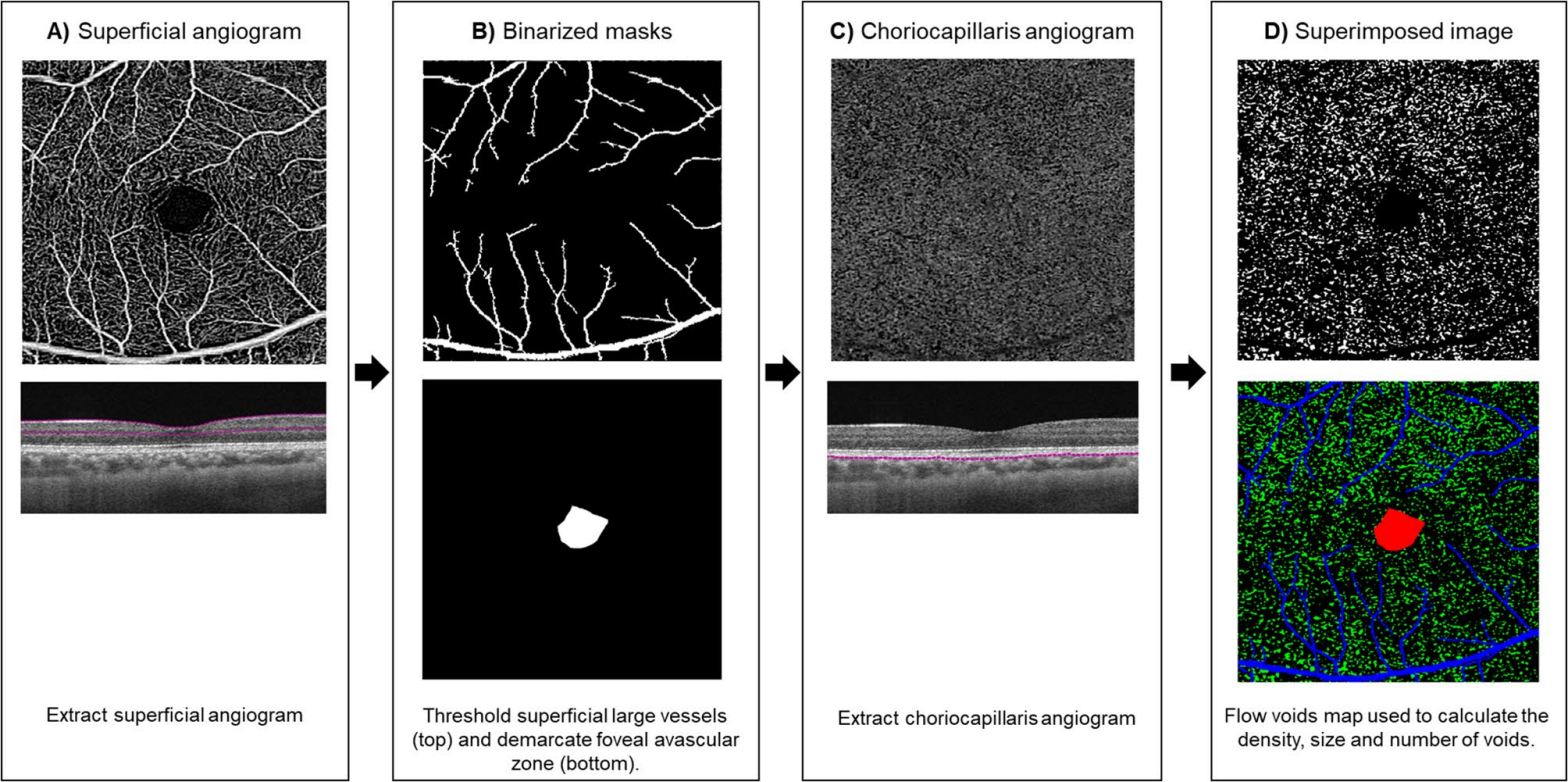
Algorithm for artefact removal and calculation of features of choriocapillaris flow voids. The angiograms and B-scans images were generated from the built-in review software (PLEX Elite Review Software, Carl Zeiss Meditec, Inc., Dublin, USA; Version 1.7.1.31492; https://www.zeiss.fr/content/dam/Meditec/international/ifu/documents/plex-elite/current/2660021169042_rev._a_artwork.pdf).

The choriocapillaris flow voids were generated based on a previously published imaging-processing algorithm using MATLAB (Fig. 4)^11^. First, superficial vascular plexus, deep vascular plexus and choriocapillaris angiography images were extracted from the OCTA device. The superficial vascular plexus image was taken from the segmentation from the inner limiting membrane (ILM) to the inner plexiform layer (IPL), the deep vascular plexus image was taken from the IPL to the outer plexiform layer (OPL) while the choriocapillaris image was taken from 31 µm below the retinal pigment epithelium (RPE) to 40 µm below the RPE. We further assessed the B-scans for any evidence of segmentation errors. Segmentation errors were defined as the incorrect identification of the retinal layers by the software. Second, projection artifacts from the overlying retinal circulation were removed from the deep vascular plexus and choriocapillaris images using the removal software that was integrated with the PLEX Elite 9,000 instrument. Projection artefacts of the overlying superficial vascular angiogram may inadvertently be included as a “flow void” if these were not removed from the calculation of flow voids. We excluded the potential noise from the flow voids calculation by excluding the large vessels from the superficial plexus and FAZ, thus providing robust data for the analysis of choriocapillaris, as compared to the other studies which did not exclude the influence of projection artefact^14,15^. Two masks from the superficial vascular plexus angiogram were created (Fig. 4B)—one was of the larger vessels^11,38^ and the other foveal avascular zone (FAZ). An intensity-based threshold was applied to generate a binarized mask of the large vessels. Third, we manually demarcated the FAZ. Afterwards, we overlaid the two masks over the choriocapillaris angiogram to remove the influence of the larger vessels and FAZ from the calculation of flow voids (Fig. 4D). We excluded the choriocapillaris flow voids measurements within the FAZ region because of two reasons. First, in healthy eyes, the flow voids in the FAZ region were less detectable than those outside the FAZ region39. Second, in myopic eyes, the flow voids tended to be more reduced within the foveal area, than the para- or peri-foveal regions^34^. Considering that all our participants are myopic, we wanted to perform the analysis that would be less affected by myopia and more generalizable to general population. Fourth, the flow voids were calculated using the 1 standard deviation (SD) thresholding strategy^12^. The flow voids were calculated as the non-perfused area divided by the area of the image excluding the large vessel and FAZ areas. The total number of flow voids were also counted, and average sizes computed as the total sizes of flow voids divide by the total number. A single flow void is defined as an unconnected object in the binarized choriocapillaris image. All flow voids analyses were evaluated for 14 subjects, where one subject has been excluded because of poor image quality.

To obtain the retinal perfusion densities of the superficial and deep vascular plexuses, we applied Frangi vessel filter^40^ to enhance the contrast of vessels, and then applied a global thresholding technique to binarize the images (Supplementary Figure S1)^41^. We then calculated the retinal perfusion densities of the superficial and deep vascular plexuses as the ratio of the segmented vessel area over the total image area. Large vessel perfusion density was segmented using a single thresholding and calculated as the ratio of the segmented large vessels over the total image area using the generated binarized mask shown in Fig. 4B.

### Statistical analyses

Intraclass correlation coefficient (ICC) was performed for intra-grader and intergrader reliability. The diurnal variation of retinal thickness, choroidal thickness, choriocapillaris flow voids variables (density, size and numbers) and retinal perfusion densities were assessed with repeated-measures analysis of variance (ANOVA), where the Greenhouse–Geisser adjustments were applied depending on the validity of the assumptions by Mauchly’s Sphericity, and within-subject factor (time of visit) was adjusted^5,6^. All statistical analysis was performed using IBM SPSS commercial analytical software (IBM SPSS statistic 23).

## Supplementary information

Supplementary information

## Data Availability

The datasets generated during and/or analyzed during the current study are not publicly available due to the terms of consent to which the participants agreed but are available from the corresponding author on reasonable request.
