# Supplementary material for: Are choriocapillaris flow void features robust to diurnal variations? A swept-source optical coherence tomography angiography (OCTA) study
Source: Sci Rep. 2020 Jul 9;10:11249. doi: 10.1038/s41598-020-68204-x (PMC7347889; doi:10.1038/s41598-020-68204-x)

Are choriocapillaris flow void features robust to diurnal variations? A swept-source optical coherence tomography angiography (OCTA) study

Emily Lin, MSc,[1*] Mengyuan Ke, MSc,[1*] Bingyao Tan, PhD,[1-3] Xinwen Yao, PhD,[1-3] Damon Wong, PhD,[1-3] Lirong Ong, BSc,[1] Leopold Schmetterer, PhD,[1-7] Jacqueline Chua, BOptom, PhD,[1,2,7]

[1] Singapore Eye Research Institute, Singapore National Eye Centre, Singapore
[2] SERI-NTU Advanced Ocular Engineering (STANCE), Singapore, Singapore
[3] Institute for Health Technologies, Nanyang Technological University, Singapore
[4] Department of Clinical Pharmacology, Medical University Vienna, Vienna, Austria
[5] Center for Medical Physics and Biomedical Engineering, Medical University Vienna, Vienna, Austria
[6] Institute of Molecular and Clinical Ophthalmology, Basel, Switzerland
[7] Academic Clinical Program, Duke-NUS Medical School, Singapore

*Shared first authorship

Word count: Manuscript: 3,649 **Abstract:** 200 **Tables:** 2 **Figures:** 4

Corresponding author: Dr Jacqueline Chua
20 College Road, The Academia, Level 6, Discovery Tower, Singapore 169856
Tel: +65 6576 7246, Fax: +65 6225 2568
Email: jacqueline.chua.y.m@seri.com.sg

Supplementary Table S1. Study design and procedures

Study Procedures/Visit Schedule		1st Visit					2nd Visit				
		9:00 am	11:00 am	1:00 pm	3:00 pm	5:00 pm	9:00 am	11:00 am	1:00 pm	3:00 pm	5:00 pm
1	Blood pressure	x	-	-	-	-	x	-	-	-	-
2	Ocular biometry	x	-	-	-	-	x	-	-	-	-
3	Auto-refraction	x	-	-	-	-	x	-	-	-	-
4	Intra-ocular pressure	x	-	-	-	-	x	-	-	-	-
5	Fundus photography	x	-	-	-	-	x	-	-	-	-
6	Swept-source optical coherence tomography angiography	x	x	x	x	x	x	x	x	x	x

Supplementary Figure S1. Algorithm to calculate the retinal vessel densities from the superficial and deep vascular plexuses.

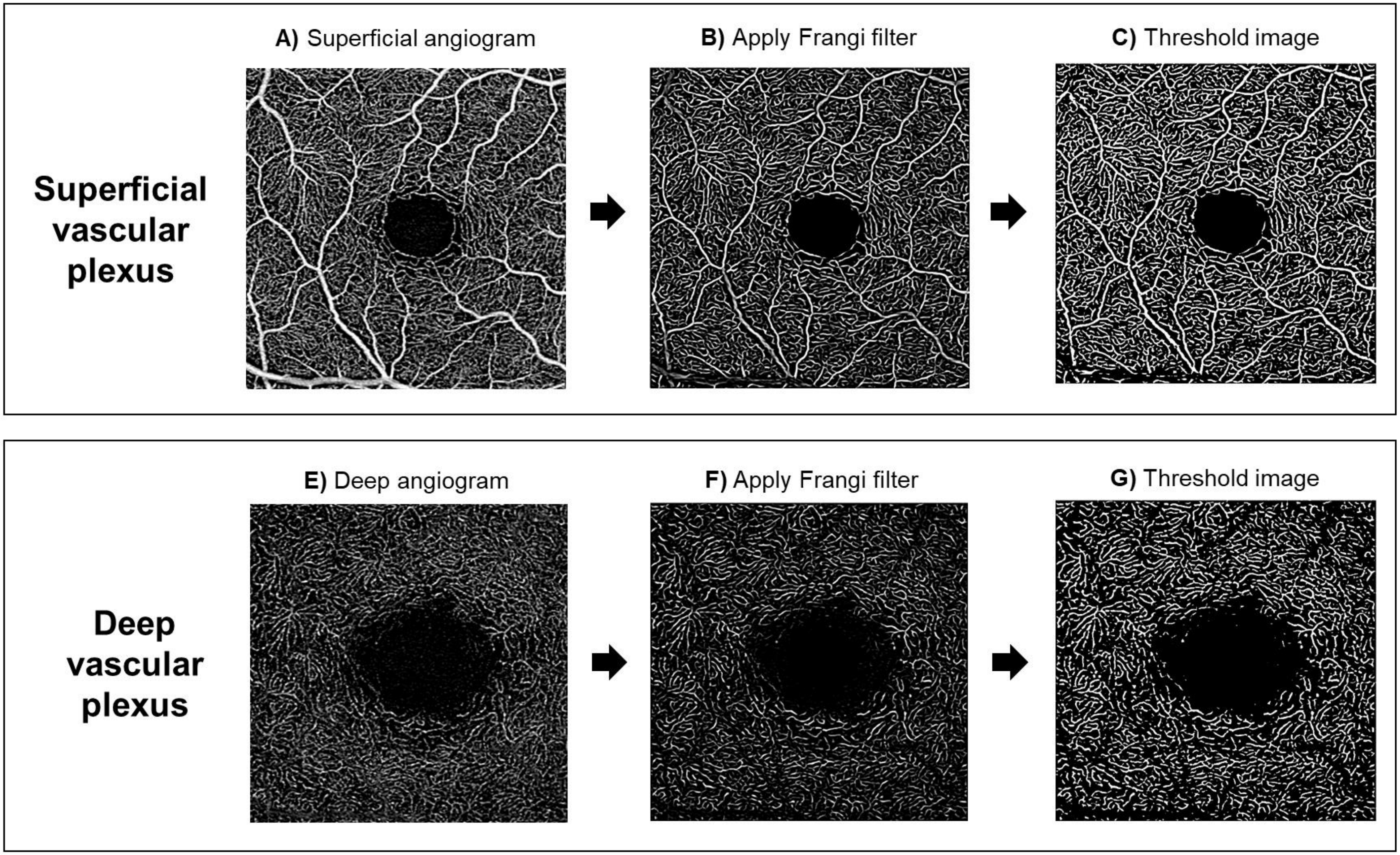

Supplement: Supplementary file 1 — Supplementary information [file 41598_2020_68204_MOESM1_ESM.pdf]
